# Aging syndrome genes and premature coronary artery disease

**DOI:** 10.1186/1471-2350-6-38

**Published:** 2005-10-31

**Authors:** Adrian F Low, Christopher J O'Donnell, Sekar Kathiresan, Brendan Everett, Claudia U Chae, Stanley Y Shaw, Patrick T Ellinor, Calum A MacRae

**Affiliations:** 1 Cardiovascular Research Center and Cardiology Division, Massachusetts General Hospital, and Harvard Medical School, Boston, MA, USA; 2 Cardiology Division, Massachusetts General Hospital, and Harvard Medical School, Boston, MA, USA; 3 The Framingham Heart Study, Framingham MA, and the National Heart, Lung, and Blood Institute, National Institutes of Health, Bethesda, MD, USA

## Abstract

**Background:**

Vascular disease is a feature of aging, and coronary vascular events are a major source of morbidity and mortality in rare premature aging syndromes. One such syndrome is caused by mutations in the lamin A/C (*LMNA*) gene, which also has been implicated in familial insulin resistance. A second gene related to premature aging in man and in murine models is the *KLOTHO *gene, a hypomorphic variant of which (KL-VS) is significantly more common in the first-degree relatives of patients with premature coronary artery disease (CAD). We evaluated whether common variants at the *LMNA *or *KLOTHO *genes are associated with rigorously defined premature CAD.

**Methods:**

We identified 295 patients presenting with premature acute coronary syndromes confirmed by angiography. A control group of 145 patients with no evidence of CAD was recruited from outpatient referral clinics. Comprehensive haplotyping of the entire *LMNA *gene, including the promoter and untranslated regions, was performed using a combination of TaqMan^® ^probes and direct sequencing of 14 haplotype-tagging single nucleotide polymorphisms (SNPs). The KL-VS variant of the *KLOTHO *gene was typed using restriction digest of a PCR amplicon.

**Results:**

Two SNPs that were not in Hardy Weinberg equilibrium were excluded from analysis. We observed no significant differences in allele, genotype or haplotype frequencies at the *LMNA *or *KLOTHO *loci between the two groups. In addition, there was no evidence of excess homozygosity at the *LMNA *locus.

**Conclusion:**

Our data do not support the hypothesis that premature CAD is associated with common variants in the progeroid syndrome genes LMNA and KLOTHO.

## Background

CAD is the most common cause of death in the developed world and an increasingly important cause of mortality in the developing world. The dominant pathophysiologic paradigm in CAD is that observed in a relatively rare inherited form of atherosclerosis; Familial Hypercholesterolemia caused by mutations in the LDL receptor[1]. In this condition a primary endocytic abnormality leads, at least partly though excess elevations in LDL cholesterol, to focal endothelial injury, and chronic inflammatory lesions of the arterial wall. Ultimately through plaque rupture and thrombosis there is episodic end-artery occlusion. Importantly, while the later phases of this process appear to be relevant to many forms of CAD, the earliest injury is unknown in most forms of vascular disease precluding truly preventative strategies. Recently the identification of Mendelian forms of coronary disease has suggested that other major pathways may contribute to each stage of the process[2,3]. Family history is a major risk factor for premature CAD, but the genetic contributions to common forms of the disease are unknown[4]. The high prevalence of CAD in older age groups suggests that some forms of atherosclerosis are an integral part of the aging process, and inherited premature aging (or progeroid) syndromes are associated with extensive vascular disease[5]. The causal genes underlying two such rare Mendelian forms of aging recently have been identified.

Discrete mutations in the *LMNA *gene have been demonstrated to cause a range of inherited syndromes including a variant of Emery-Dreifus muscular dystrophy, dilated cardiomyopathy with conduction disease and Charcot-Marie-Tooth disease[6]. The mechanisms of this remarkable pleiotropy are unknown, but have been attributed to discrete functions of different lamin domains in individual tissues. At least two of the syndromes caused by lamin mutations include premature CAD. Some lamin mutations result in Dunnigan's partial lipodystrophy in which CAD is a prominent feature, especially in females. These individuals suffer from an unusual form of insulin resistance, with morphologic abnormalities including hemifacial loss of subcutaneous adipose tissue, as well as hypertension, dyslipidemia and vascular disease. Patients in such families also have elevated CRP levels, and lower leptin and adiponectin levels[7]. A second aging disorder, Hutchison-Gilford Progeria Syndrome (HGPS), is the result of recurrent *de novo *mutations of a single nucleotide in exon 11 of the *LMNA *gene have been shown to cause the progeroid disorder)[8,9]. HGPS is characterized by extreme 'aging' in multiple tissues with most affected individuals dying from atherosclerotic vascular disease in their late teens. In many cases of HGPS there also appeared to be germ-line loss of the second *LMNA *allele, suggesting that somatic mutations at this locus might lead to more common forms of aging, possibly in a tissue-restricted manner. Given the extreme forms of vascular disease seen in these two laminopathies, and the strong association of the metabolic syndrome with CAD we explored the role of *LMNA *variation in premature coronary disease.

The second gene implicated in progeria is *KLOTHO *encoding a membrane protein of unknown function sharing homology with beta-glucosidases. Targeted deletion of the *Klotho *locus in mice results in reduced longevity, vascular disease, osteoporosis and chronic lung disease[10]. Recent work has demonstrated impaired angiogenesis and vasculogenesis in these *Klotho*-deficient mice[11]. A role for the ortholog of *Klotho *in human aging was suggested by the finding that a specific *KLOTHO *allele (KL-VS) which changes amino-acid sense is underrepresented in older age groups. This finding was reproduced in three ethnically distinct groups[12]. Individuals homozygous for the KL-VS allele were 2.6-fold less likely to survive to 65 years of age or greater. A role for the human *KLOTHO *gene in vascular disease also has been suggested by work demonstrating that the same KL-VS allele is associated with increased risk of occult atherosclerosis in a high-risk sample consisting of siblings of individuals with premature CAD. This effect of the KL-VS allele was evident even after adjustment for known risk factors [13]. We also explored the role of the KL-VS allele in our cohort of subjects with premature CAD.

Previous studies of these two aging genes largely have been confined to rare kindreds or cohorts with direct evidence of CAD. We tested the hypothesis that common variants at the *LMNA *and *KLOTHO *loci were associated with angiographically-defined premature CAD in a case-control association study.

## Methods

### Subjects

Between 1999 to 2003, we attempted to recruit serial patients with premature CAD presenting to the Massachusetts General Hospital were recruited. Inclusion criteria were; documented acute coronary syndrome (myocardial infarction or unstable angina) confirmed on coronary angiogram; and age ≤ 50 years for males or ≤ 55 years for females. Exclusion criteria were: logistic difficulties precluding enrollment, history of familial dyslipidemia, type I diabetes, endstage renal disease, a history of recent trauma, sepsis, or previous thoracic irradiation. All patients gave written informed consent prior to study enrollment. A structured interview and physical examination was performed during the initial hospitalization. This included a detailed medical history, careful documentation of cardiovascular risk factors, current and past medications and a comprehensive family history using an instrument which we have previously validated. Hypertension was defined as previous antihypertensive use or a documented untreated systolic blood pressure >140 mmHg or diastolic>90 mmHg. Hypercholesterolemia was defined as an LDL cholesterol >130 mg/dl. Diabetes mellitus was defined as the use of diet, oral hypoglycemic agents or insulin to control blood glucose. Smoking was defined as the regular use of tobacco at any stage in the previous decade. A blood sample for nucleic acid extraction was obtained at enrollment. In parallel, over the same time period a control population was recruited from outpatient referral clinics at Massachusetts General Hospital. These control subjects were free from symptoms suggestive of CAD or ECG abnormalities, and all had undergone transthoracic echocardiography to exclude the presence of subclinical structural heart disease.

### Genetic analyses

#### *LMNA *genotyping

To generate representative haplotypes at the *LMNA *locus, we first assembled *in silico *35,374 base pairs of reference sequence encompassing the entire coding region of the gene including sequence reported by Lin et al[14], consensus finished sequence and trace data from the region from the Human Genome Project[15]. Additional direct sequencing of the relevant genomic regions was performed where necessary. Sequence assembly and analysis was performed using the aid of Vector NTI version 8 (InforMax™). SNPs spanning the *LMNA *gene were selected from dbSNP [16] and the published literature. Selection was based on genomic coverage and where possible on polymorphism information content. In addition, all common (defined as those present in ≥ 5% of the population) SNPs changing amino acid sense were included.

A combination of TaqMan^® ^based assays and direct sequencing was used to type the SNPs in an initial subset of the study cohort. Details of probes and primers are available in Table 5. Allelic discrimination using TaqMan^® ^was performed using 5 ng of sample DNA in a 25 μL reaction containing 12.5 μL TaqMan^® ^Universal PCR Mix (Applied Biosystems), 300 nM primers, 200 nM TaqMan^® ^MGB probes (Applied Biosystems). Reaction conditions consisted of preincubation at 50°C for 2 minutes, 95°C for 10 minutes, then cycling for 40 cycles of 95°C, 15 seconds; 60°C, 1 minute. Amplifications were performed in an ABI Prism 7000 machine (Applied Biosystems) for continuous fluorescence monitoring. Direct cycle sequencing was used to type one series of closely linked SNPs as detailed in Table 5.

For each *LMNA *SNP the allele frequencies were defined and testing for Hardy Weinberg equilibrium was performed. Haplotypes at the LMNA locus were defined using the modified estimation-maximization algorithm implemented in the software package Haploview [17]. Following confirmation that specific SNPs did not segregate independently but were in linkage disequilibrium with each other, the methods of expectation-maximization-based haplotype frequency estimation and permutation-based hypothesis testing were performed as previously described [18].

#### *KLOTHO *genotyping

The KL-VS allele was typed using modifications of the published conditions[12]. Sample DNA was amplified by PCR (sense primer 5'-GCCAAAGTCTGGCATCTCTA-3'; antisense primer 5'-TTCCATGATGAACTTTTTGAGG-3') under the following conditions: 95°C for 2 minutes, followed by 35 cycles of 94°C for 30 seconds, 60°C for 30 seconds, and 72°C for 1 minute, followed by a 20 minute 72°C final extension. PCR products were then digested with *Mae*III (Roche) at 55°C for 16 hours and electrophoretically separated on a 2% agarose gel. The KL-VS allele is characterized by diagnostic *Mae*III restriction fragments of 265 and 185 bp respectively. Allele frequencies were determined by gene counting.

### Statistical analyses

Continuous variables are presented as means ± SD. Baseline characteristics were compared using the Student's unpaired *t *test for continuous data and the Chi-square or Fisher's exact test for categorical data. Single-locus tests of association between either SNP allele frequencies or SNP genotype frequencies and case-control status were carried out using standard contingency Chi-square tests. Based on the method of Chapman and Nam and assuming an allele frequency of 0.20, our study design has 93% power to detect a difference in allele frequency of 1.8 times or more at α = 0.05 [19].

## Results

### Subjects

During the study period we enrolled 295 subjects with premature CAD and 145 controls. The premature CAD cohort, as expected, exhibited a higher proportion with diabetes mellitus, hypertension, and smoking (see Table 1). In addition, the premature CAD cohort was younger and more likely to be obese. Self-reported ethnicity was similar between the two cohorts.

### *LMNA *genotypes

**Figure 1:**
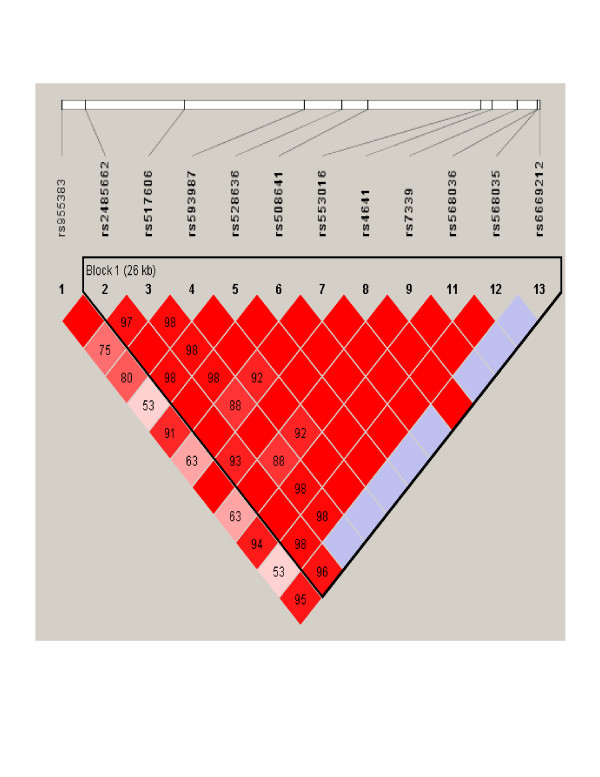
Linkage disequilibrium plot demonstrates the inheritance of tagged SNPs as a single block within the *LMNA *gene.

Two SNPs (rs3204564 and rs536857) that were not in Hardy Weinberg equilibrium were not included in subsequent analyses. Median spacing of the SNPs was 1,206 bp apart with a range of 146 bp to 7,063 bp (SNPs rs568035 and rs568036 are adjacent to each other). Haplotype analysis revealed the presence of 6 haplotypes, with the most frequent occuring at an overall frequency of 43.8% (see Figure 1 and Table 2). Haplotype allele frequencies did not differ significantly between patients and controls. Subsequently each individual SNP was tested independently for association with premature CAD. No significant difference in allele frequency for any SNP in the *LMNA *gene was observed between the control and patient groups (see Table 3). In analyses adjusting for age, BMI, gender, hypertension, diabetes mellitus, statin use, and smoking history we did not observe any significant associations.

### *KLOTHO *genotypes

Previous studies have documented the heterozygote carrier frequency for the KL-VS allele to be between 20 to 30% and the homozygosity frequency to be between 1 to 4% [12]. The allele distribution is very similar in our patient and control cohorts, and no significant difference in allele frequencies was observed between these groups (see Table 4). The frequency of the KL-VS allele was 14.0% in patients with premature CAD compared to 18.2% in the control population (p = 0.09).

## Discussion

We hypothesized that common variation at the *LMNA *or *KLOTHO *loci might result in typical forms of coronary disease in the absence of progeroid syndromes. We tested not only the hypothesis that alleles of *LMNA *or *KLOTHO *would be associated with premature CAD (a population enriched for inherited contributions), but also pre-specified that such premature vasculopathy might be associated with an excess of homozygosity at the *LMNA *locus. Our data suggest that, within the constraints of the current study, there is no association between common variation in the aging genes *LMNA *or *KLOTHO *and rigorously defined premature CAD. Further, there was no evidence of excess homozygosity at the *LMNA *locus, rendering a somatic "two-hit" mechanism at this locus less likely as a potential cause of CAD. These data contradict previous findings in smaller studies and emphasize the difficulties intrinsic to such genetic association studies.

### Possible explanations for contradictory findings

Genetic association studies relating common or "complex" phenotypes in large patient cohorts may be the only method capable of unraveling small population-wide genetic effects, but these studies prove difficult to reproduce and are of limited utility in defining causation [20-24]. Several intrinsic limitations of genetic association approaches contribute to the disparity between our results and previous studies of the *LMNA *and *KLOTHO *loci including the low prior probability of any observed association, population stratification, and varying degrees of linkage disequilibrium with neighbouring genes [20,25]. One of the most difficult potential confounders is underlying etiologic heterogeneity, magnified by the relatively low resolution of many traditional clinical phenotypes[24]. Clearly, not all CAD is caused by the same mechanism, and there is variation in the biologic behaviour of the various syndromes, ranging from occult chronic ischemia through to ischemic sudden death. The heritable basis for each of these components in the "spectrum" of CAD may be quite distinct. This phenotypic heterogeneity remains an important issue despite our rigorous use of coronary angiographic diagnoses. Our control population was older and had a male predominance, both known cardiovascular risk factors. The patient population had a higher prevalence of diabetes mellitus, hypertension, and smoking. This is not unusual as most patients with CAD already have documented cardiovascular risk factors [26], and it is conceivable that many 'risk factors' are actually manifestations of subclinical forms of vascular disease. By excluding patients presenting with undifferentiated chronic stable CAD we hoped to minimize the phenotypic heterogeneity. Nevertheless, it remains possible that the progeroid genes we have studied are associated with a particular subset of CAD, but not with premature disease presenting as acute coronary syndromes.

Additional factors also may explain our findings. Our study would not have detected somatic mutations present only in the vessel walls. While this is relatively unlikely given the common progenitors shared by hematologic and endothelial lineages, mutations restricted to more differentiated cells may not be detectable. The association between the *KLOTHO *gene and premature atherosclerosis seen in the study by Arking, et al.[13] may reflect a chance relationship between reduced survival from other causes and a common trait, but by studying acute syndromes we may have selected a distinct subset of disease in which the effects of *KLOTHO *have been diluted.

### Study limitations

Our study has several intrinsic limitations. The control population did not undergo invasive clinical testing to definitively exclude CAD, but nonetheless had extensive non-invasive evaluations including echocardiography. The genotype frequencies observed suggest that the current study is adequately powered to detect a risk ratio of 1.8 or more [22], but would be unlikely to detect smaller population wide effects or large effects from rare alleles. The contributions of rare alleles would be better addressed using a family based strategy[27].

### Future genetic studies in CAD

Association studies remain controversial and our current study demonstrates some of the problems encountered with this approach. Despite rigorous phenotyping, detailed haplotyping and adequate power to detect a genetic effect of similar magnitude to that seen in previous studies of CAD, we did not see any significant differences in genetic architecture between our study and control populations. We have therefore demonstrated that genes associated with the progeroid syndrome are not likely to have a major effect on the development of premature atherosclerosis, despite a clear biological rationale.

In spite of the heterogeneity of CAD, insights from rare Mendelian variants have proven broadly applicable. The identification of new pathways through such familial forms of CAD will contribute to our understanding of all forms of vascular disease[2,3]. Linkage based family analyses are likely to yield more robust results than association studies and we believe increasingly will be used in future studies on the genetics of CAD. Some disorders undoubtedly result from more common ancient alleles, and understanding the basic haplotype structure of the human genome will facilitate their identification[28]. However, the genetic dissection of common conditions will require much more finely textured phenotypes than those traditionally employed in clinical medicine[24].

## Conclusion

Common variants in aging syndrome genes previously implicated in CAD are not associated with rigorously defined premature acute CAD. This negative finding may reflect the specific phenotypes tested and highlights one of the major limitations of genetic association studies, the phenotypic heterogeneity of most 'common' diseases.

## Abbreviations

CAD-coronary artery disease

LMNA-lamin A/C

HGPS-Hutchison Gilford Progeria Syndrome

SNP-single nucleotide polymorphism

## Conflict of interest

The author(s) declare that they have no competing interests.

## Authors' contributions

AFL was involved in the study design, carried out the molecular genetic studies, participated in the sequence analysis and drafted the manuscript. SK, PTE, CUC, SYS, BE and CAM, participated in the recruitment of subjects. CAM and COD participated in the design of the study and performed the statistical analysis. CAM and COD conceived of the study, and participated in its design and coordination and helped to draft the manuscript. All authors read and approved the final manuscript.

## Pre-publication history

The pre-publication history for this paper can be accessed here:



## Figures and Tables

**Table 1:** Baseline demography of study cohorts. Values are presented as number (percentage) unless otherwise indicated.

**Baseline Characteristics**	**PCAD**	**Controls**	**P-value**
Number	295	145	
Age at enrollment, years	47.4 ± 7.1	54.3 ± 10.8	<0.001
Male gender	199 (68.2)	115 (79.3)	
BMI	30.2 ± 6.6	27.1 ± 4.4	<0.001
**Cardiovascular risk factors**			
Diabetes mellitus	55 (18.8)	7 (4.86)	<0.001
Hypertension	112 (38.2)	15 (10.4)	<0.001
Hypercholesterolemia	104 (35.4)	30 (38.0)	0.67
Statin use	104 (35.4)	30 (38.0)	0.67
Smoking	113 (38.4)	8 (12.5)	<0.001

**Table 2:** Distribution of major haplotype blocks in LMNA among patients with premature CAD and the control population.

Haplotype												Frequency			
Haplotype	i	ii	iii	iv	v	vi	vii	viii	ix	x	xi	Overall	PCAD*	Controls	p-value

I	C	G	G	A	T	G	C	G	A	C	G	0.44	0.43	0.45	0.55
II	C	G	G	A	T	G	T	G	A	C	G	0.30	0.29	0.29	0.85
III	T	G	G	A	T	G	C	G	A	C	A	0.13	0.13	0.14	0.82
IV	T	T	A	G	C	A	C	C	G	T	G	0.05	0.05	0.07	0.25
V	T	G	G	A	T	G	C	G	A	C	G	0.05	0.05	0.04	0.41
VI	T	G	G	G	T	A	C	C	A	T	G	0.02	0.02	0.01	0.23

SNP ids; i-rs2485662;ii-rs517606;iii-rs593987;iv-rs528636;v-rs508641;vi-rs553016;vii-rs4641;viii-rs7339;ix-rs568036;x-rs568035;xi-rs6669212.

*PCAD: Group with premature coronary artery disease

2 SNPs (rs3204564 and rs536857) that were not in Hardy Weinberg equilibrium were excluded.

**Table 3:** Frequency of major SNP allele in *LMNA *among patients with premature CAD and the control population.

***SNP***	***Major Allele***	Freq in PCAD*	***Freq in Controls***	***Minor Allele***	***p-value***
rs955383	A	0.78	0.76	G	0.46
rs2485662	C	0.73	0.74	T	0.75
rs517606	G	0.94	0.93	T	0.55
rs593987	G	0.94	0.93	A	0.62
rs528636	A	0.92	0.92	G	0.92
rs508641	T	0.95	0.93	C	0.35
rs553016	G	0.93	0.92	A	0.89
rs4641	C	0.70	0.71	T	0.81
rs7339	G	0.93	0.92	C	0.90
rs568036	A	0.95	0.93	G	0.35
rs568035	C	0.92	0.92	T	1.00
rs6669212	G	0.86	0.86	A	1.00

* PCAD: Group with premature coronary artery disease

2 SNPs (rs3204564 and rs536857) that were not in Hardy Weinberg equilibrium were excluded.

**Table 4:** Genotype frequency of *KLVS *in cohort

***Genotype***	PCAD*	***Frequency***	***Controls***	***Frequency***	***Chi-square***	***p-value***
WW	216	73.5%	95	66.4%	2.321	0.13
WK	73	24.8%	44	30.8%	1.731	0.19
KK	5	1.7%	4	2.8%	0.574	0.45
Total	294		143			

* PCAD: Group with premature coronary artery disease

**Table 5:** Analysis of SNPs in *LMNA*. Details of primers and probes used in TaqMan^® ^analysis and PCR conditions for direct sequencing.

**SNPs typed by TaqMan^®^**					
*SNP ID*	*Position*	*Forward Primer (5'-3')*	*Reverse Primer (5'-3')*	*TaqMan^® ^probe (VIC)*	*TaqMan^® ^probe (FAM)*

rs955383	2,533	GGCCAGGAGTTTTAGACCAAATT	TGAGTAGCTGAGACTATAGGCTATCATG	ATAGCAAGgCTCCGTC	TAGCAAGaCTCCGTCTC
rs2485662	3,971	ACTACCTTCTTTCTGGCTGAAACCAG	GGGGAAGCAGGGCTGGG	AGACCCAAtATTGGCT	ACCCAAcATTGGCT
rs517606	9,827	CGAACTCCTAGGCTCAAGTAATCC	GGGCTACAGACTAGAAAGAGACAGAGA	CTGGGATgTATAGGCA	CTGGGATtTATAGGCATGA
rs593987	16,890	TTGCTGTGCTGGTGCCTTT	GAGTTGGCACTTGCAAATGTGA	AGCCgGACTTCCT	AGCCaGACTTCCTTG
rs528636	19,123	TCTAAATTCTGAGAGCCTCCTAGTACA	GCAACTTAGATTCCGAGCTCCTT	AGCAGCCaTTAGC	AGCAGCCgTTAGC'
rs508641	20,642	GGAGAGAGAGGGAAAAGCATTCA	TGACTCAGGGCTCAGGAACGT	ACGGGGtAGAGCT	ACGGGGcAGAGCT
rs553016	27,366	GCTTGGGACTCTGGGGAG	CTTCCACACCAGGTCGGTA	TCCCATCgCCACCCA	TCCCATCaCCACCCA
rs4641	28,037	CGAGGATGAGGATGGAGATGA	TCAGCGGCGGCTACCA	TCCATCACCACCAcGTG	CCATCACCACCAtGTG
rs7339	29,479	CAGAACTGCAGCATCATGTAATCTG	GGGTTATTTTTCTTTGGCTTCAAG	CCTGCACgTCATGG	CCTGCACcTCATGG
rs3204564	30,139	AGGTGGAAGAAGGGAGAAGAAAG	GCACCCCACTTGGCTTCA	CCTAGCTTTAgACCCTGG	CCTAGCTTTAaACCCTGG
rs536857	31,790	CACTGTGGGCTGGGGAACAC	CCTCAGCCCTCCTCCTCAAGAG	AGCAGGCaACGTT	AGCAGGCgACGTT

**SNPs typed by direct sequencing**					

*SNP ID*	*Position*	*Primers(5'-3')*	*PCR conditions*		

rs568036	30,669	These SNPs are contained in the amplicon amplified by the primers 5'-ACTGCATCCTCCTGCTCATT-3' and 5'-GGCTCCTACTTGGCCTAACA-3'	95°C for 2 min, followed by 35 cycles of 94°C for 30 s,60°C for 30 s, and 72°C for 90 s, followed by a 20 min 72°C final extension.		
rs568035	30,670				
rs6669212	30,816				
